# Occurrence of Fungi and Mycotoxins in Fish Feeds and Their Impact on Fish Health

**DOI:** 10.1155/2019/6743065

**Published:** 2019-11-11

**Authors:** Esther Marijani, Emmanuel Kigadye, Sheila Okoth

**Affiliations:** ^1^Open University of Tanzania, P.O. Box 23409, Dar es Salaam, Tanzania; ^2^University of Nairobi, School of Biological Science, P.O. Box 30197-00100, Nairobi, Kenya

## Abstract

The rapid population growth in developing countries has led to strong pressure on capture fisheries. However, capture fisheries have reached their maximal limits of fish production and are supplemented by farmed fish. The growth in aquaculture has led to high demand for fish feeds, which play a very important role in fish nutrition and health. Use of animal protein in fish feeds is expensive; hence, a majority of farmers from developing countries use local feed ingredients from plant origin as a source of dietary protein. However, these ingredients of plant origin provide the best natural substrates for fungi, which can be easily accompanied by mycotoxin development under suitable conditions. The locally made feed comprises ingredients such as soybeans, cottonseed cake, and wheat and maize bran which are mixed together and ground after which the compounded feed is pelleted and stored. Among the ingredients, maize and oilseeds are more susceptible for mycotoxigenic fungi compared to other ingredients. The outcomes of mycotoxin contamination in fish feeds are not different from other animal species intended for human consumption, and they are directly associated with production losses, particularly decreased weight gain and feed conversion, impaired immune system and reproductive performance, and increased fish mortality. Fish may also carry mycotoxin residues along the food chain, thus compromising human health. Hence, it is important to ensure the control of mycotoxin contamination in fish feeds, especially during the production and storage.

## 1. Introduction

Fish production in 2012 surpassed 158 million metric tons, whilst the value of international fish trade added up to USD 129 billion [1]. Aquaculture is the world's fastest growing food production sector for more than four decades, whereas an increasingly large share of fish entering the global markets is derived from aquaculture [2]. Aquaculture production in Africa significantly increased from 646,182 to 1,710,910 million metric tons; as a matter of fact, fish and shrimp production in Africa has already grown by approximately 400% [3]. This rapid increase has been due to a decline in fish production from capture systems and increased awareness of the nutritional importance of fish [4]. With the rapid growth in aquaculture, demand for fish feeds is expected to continue increasing proportionately [5].

Fish feed is an essential part of the aquaculture industry and comprises 40–50% of the total production cost in intensive culture systems [6]. It also has an important contribution to the production of fish feed since it tends to affect the quality of eggs [7]. A majority of fish farmers in developing countries use locally made fish feeds or commercially imported feeds for Nile tilapia (*Oreochromis niloticus)* and African catfish (*Clarias gariepinus*) production. The imported fish feed is more expensive than the locally made ones. Hence, many fish farmers use locally made fish feed usually produced and stored in large quantities in order to reduce production costs and increase profit margins [8]. The locally made feeds are usually from locally available plant and animal wastes like rice, maize and wheat bran, blood meal, cottonseed cake and sunflower seedcakes, and soybeans and cassava [9]. These ingredients can be ground manually and mixed in a hand-operated mixer and then made into pellets using a pelleting machine. Such ingredients are often subjected to contamination by molds during preharvest and/or due to poor storage conditions [10]. Prolonged storage and high temperature and humidity conditions are some of the factors that facilitate fungal development and production of attendant mycotoxins, compromising feed quality that can adversely affect the health of animals and humans [11].

Some molds are capable of producing mycotoxins, and some of these mycotoxins can cause some degree of acute toxicity when given in high amounts and are the potential carcinogens [12]. *Aspergillus*, *Fusarium*, and *Penicillium* are the three most important genera of toxigenic fungi in the tropics [13]. The presence of toxigenic fungi, some producing mycotoxin in farmed fish has increased in recent years owing to the increasing use of plant materials as components for fish feeding [5]. Contamination of fish feeds by mycotoxins and the possible transfer of these toxins into farmed fish and fish-derived products for human consumption remain a serious food safety concern [14].

Around 300–400 mycotoxin types are known to date [15], but the most important in tropical countries are aflatoxins (AFs) (AFB_1_, B_2_, G_1,_ and G_2_) and fumonisins (FBs) (FB_1_, FB_2,_ and FB_3_) [16]. In addition to AFs and FBs, ochratoxin A (OTA) and trichothecenes (THs) are also important [17].

Mycotoxin contamination has been implicated with a reduction in fish productivity, anemia, hemorrhaging, liver impairment, weight loss, increased vulnerability to secondary infectious diseases, reduced reproductive capacity, and even mortality [18–21], resulting in serious economic losses [22, 23].

Since aquaculture is a growing sector in a majority of the developing countries, especially in Africa, the aim of this review is to give an overview of fungal and mycotoxin contamination of fish feed, feed ingredients, and their effects on fish health.

## 2. Feed Consumption in Farmed Fish

Fish feed is the major cost item in intensive farming systems, and they represent 50 to 70 percent of fish farmers' production costs [24]. Requirements for intensive aquaculture are high-quality animal protein, lipid, and other essential nutrients [25]. In order to reduce the feed cost, several efforts have been made to replace the expensive feed ingredients. Incorporating vegetable oil, increasing levels of plant ingredients, and reduction in the level of costly fish meal are appropriate husbandry practices that have been adopted to reduce maintenance costs (particularly feed costs). Zhao et al. [26] reported that fish meal can be completely replaced by soy protein concentrate by increasing feeding frequency for Nile tilapia less than 2 grams. A combination of 76% rice bran and 24% fish meal, which is a mix of dried freshwater shrimp *Caridina* sp., or maize bran, sometimes with the addition of some “*dagaa*” *Rastrineobola argentea* meal, shows good growth performance on fish [8, 27]. Despite their higher nutritional values, plant ingredients in fish feeds have a higher potential of being contaminated with toxigenic fungi than animal ingredients. This potential is further increased in tropical and subtropical conditions due to storage under humid and hot conditions [28].

## 3. Occurrence of Mycotoxigenic Fungi in Fish Feeds and Ingredients

Fungi are ubiquitous in the environment, being found in water and suitable organic nutrients when appropriate temperature conditions prevail [17]. They have been reported to occur in food and feed worldwide with some of them capable of producing a wide array of mycotoxins. Four major genera stand out: *Alternaria*, *Aspergillus*, *Fusarium*, and *Penicillium* [17]. Among the genus *Aspergillus*, *A. flavus* and *A. parasiticus* are the major aflatoxin producers that are likely harmful to animals or humans [11]. Several *A. flavus* strains may or may not produce either aflatoxin B_1_ or B_2_ [11]. Secondary metabolites such as sterigmatocystin, cyclopiazonic acid (CPA), kojic acid, beta-nitropropionic acid, aspertoxin, aflatrem, and aspergillic acid are other toxic compounds also produced by *A. flavus* [11]. *Aspergillus parasiticus* produces aflatoxins G_1_ and G_2_, in addition to aflatoxins B_1_ and B_2_, but does not produce CPA [11]. *Aspergillus ochraceus* and *A. carbonarius* are the main producers of ochratoxin A (OTA), and they are commonly found in grapes, dried vine fruits, wine, and coffee [29]. However, only a few strains of *A. niger* are capable of producing OTA [30]. *Aspergillus fumigatus* is the main producer of genotoxic and cytotoxic mycotoxins, like gliotoxin [31]. Some other species of *Aspergillus*, such as *A. tamarii* and *A. versicolor*, produce CPA and fumuclavine A and have been responsible for “kodua” poisoning [32]. Kodua is known for poisoning that causes tremors, sleepiness, and giddiness [32, 33].

*Aspergillus* species are ubiquitous in tropical environments and are predominantly isolated in food and feed from the tropics [11]. Fish feeds are frequently contaminated by *Aspergillus* species (Table 1). Several studies performed in tropical countries reported that *A. flavus* and *A. parasiticus* are the major dominant species isolated from fish feed [33, 37, 39, 40]. Fallah et al. [39] and Hassan et al. [44] found that 47.5% and 70% of fish feed from Egypt and Iran, respectively, were contaminated by *A*. *flavus.* Fish feeds from Brazil and East Africa were contaminated by *A. flavus* at 35% and 54.5%, respectively [35, 40]. *Aspergillus tamarii* were isolated at a frequency of 9.1% and 8% in fish feeds from East Africa and Iran, respectively. *Aspergillus niger* (6%, 13.9%, 36%, and 39.1%) and *A. ochraceus* (10.2%) as the potential ochratoxigenic fungi were isolated from fish feed from East Africa, Iran, Portugal, and Brazil [10, 35, 39, 40]. Other *Aspergillus* species commonly isolated from fish feeds are *A. versicolor, A. fumigatus*, *A. candidus*, *A. glaucus*, and *A. oryzae* [35, 37, 39, 40]. Table 1 compares data from previous studies on the most frequently isolated fungi from fish feeds and their ingredients.

*Fusarium* species are destructive pathogens on cereal crops and other commodities and produce some of the most commonly encountered mycotoxins including the trichothecenes and fumonisins in feeds [16]. *Fusarium verticillioides* is ubiquitous in maize and produces FBs, which are generally more prevalent when crops are under drought stress or suffer excessive insect damage [45]. *Fusarium graminearum* is the main producer of the deoxynivalenol (DON) and estrogenic compound zearalenone (ZEN) [46]. This species is pathogenic on maize, wheat, and barley and produces these toxins whenever it infects these grains before harvest. *Aspergillus niger* also produces FBs, and several commodities may be affected [30]. The most common fungi associated with maize which is the common ingredient in fish feeds are *Fusarium verticillioides* and *Fusarium proliferatum*. A study on the infections of *Fusarium* sp. in feed ingredients was done by Ivić et al. [47] and found that the dominant species were 27% of *F. graminearum* isolated on wheat, 83% of *F. verticillioides* isolated on maize, and 34% of *F. sporotrichioides* isolated on soybean. The authors of this study concluded that the risk of contamination with *Fusarium* toxins is higher for maize and wheat than for soybean. *Fusarium* species appear to be uncommon to fish feeds as they were isolated in a very small percentage but may cause adverse effects to fish [10, 37, 39, 42].

Some *Penicillium* species are mycotoxin producers and can negatively affect the health of humans and animals [48]. *Penicillium verrucosum* produces OTA but occurs only in cool temperate climates, where it infects small grains like wheat and barley [48] and not commonly isolated in tropical areas. *Penicillium citrinum* is a species where citrinin toxin was first isolated, but the toxin has subsequently been identified to be produced by several *Penicillium* and *Aspergillus* species [49]. No information is available on the contamination of citrinin in fish feeds; however, its producer *P. citrinum* has been isolated from fish feeds [37, 40].

*Penicillium expansum*, *Aspergillus, Penicillium*, and *Paecilomyces* fungal species produce patulin, whereas ergot alkaloids are compounds produced as a toxic mixture of alkaloids in the sclerotia of the species of *Claviceps* [50]. *Penicillium expansum* is particularly linked with a range of moldy fruits and vegetables, especially in rotting apples and figs while the species of *Claviceps* are common pathogens of several grass species. *Penicillium expansum* occurs best in wet, cool (<25°C) environments, and the growth rate was the fastest at a relative humidity of 90% [50]. *Penicillium expansum* has been isolated in fish feeds, and the use of vegetables as feed ingredients could be the reason for the presence of this species in the finished fish feeds [38].

*Penicillium crustosum* and *P. commune* are closely related and produce CPA and roquefortine C (ROQ-C) [51]. These species were infrequently encountered in fish feeds [38]. *Penicillium chrysogenum* is also a ROQ-C producer and had been isolated in fish feeds [35, 38]. Other *Penicillium* species which are isolated in fish feeds are *Penicillium glabrum, P. nalgiovense*, *P. corylophilum*, *P. implicatum*, and *P. restrictum* [37, 38, 41].

The genus *Eurotium* is also an important mycotoxin producer and grow exceptionally well at low water activities [52]. Most of the *Eurotium* species are of special interest to feed mycology due to their xerophilic physiology; many isolates are able to grow at water activities below 0.75, and some isolates are able to grow at values as low as 0.64 *a*_*w*_ [53]. *Eurotium amstelodami*, *E. repens*, and *E. rubrum* reported to produce aflatoxin [23, 38], and ochratoxin A is produced by *E. amstelodami* [23]. Although most of the studies on fish feed mycobiota have been concentrated on the presence of mycotoxigenic genera *Aspergillus, Fusarium*, and *Penicillium*, *Eurotium* sp. have as well reported as frequent contaminants [38, 39, 41]. Commonly, *Eurotium* species isolated from fish feeds are *E. repens* and *E. rubrum* [35, 38]. *Eurotium rubrum* were isolated with a frequency of 25% and 3%, while *E. repens* (21.4%) were isolated from fish feed from East Africa and Argentina. Since some of the *Eurotium* species produce aflatoxin and OTA, their presence in fish feeds could affect fish health and cause serious economic losses.

The commonly used raw ingredients in the manufacturing of fish feeds are maize, rice, wheat bran, soybean, sunflower seed cake, and cottonseed cake, which are highly susceptible to mycotoxin-producing fungi [17, 54, 55]. Several mycotoxigenic fungi had been isolated from ingredients used for fish feed from different fish farms around the world [28, 35, 36]. The presence of these mycotoxigenic fungi in the ingredients might also cause the formation of mycotoxin in the finished fish feeds [35, 38, 40]. *Aspergillus flavus* was the dominant fungal species isolated from maize bran, cottonseed cake, sunflower oil seed cake, and cassava; however, soybean samples were the least contaminated by mycotoxigenic fungi [35]. In another survey conducted in Brazil, soybean bran intended for fish feeds were contaminated by *A. flavus* (61.5%), *P. citrinum* (81.8%), and *A. parasiticus* (7.7%) [37]. *Aspergillus flavus* was the dominant species isolated from maize bran (50%), and other cereals (66.6%) were designated for fish feed from Brazil. Other fungal species isolated at higher frequencies from maize bran and other cereals were *P. citrinum* (50% and 90.9%) and *A. parasiticus* (16.7%), respectively [37].

Factors like high temperature and relative humidity, together with inappropriate handling and storage practices increase the likelihood of the growth of mycotoxigenic fungi. In addition, storage practices in most developing countries are poor, which make them more vulnerable to fungal contamination [56]. Also, insect infestation can cause heating and generation of moisture. For the cereal grain, an increase in temperature is expected due to respiration, which may likewise occur due to insect or fungal activity. Heating results in moisture condensation in cool areas inside the grain mass [57]. As a result of this, further fungal growth and insect infestation are encouraged. Normally, the standard moisture content required for most cereals often used as feed ingredients should be 14% before storage [58]. Therefore, it is very important to utilize feed ingredients which have moisture content at or below the safe level of 13% [58]. Aeration with cool air may also help to protect the stored ingredients against fungal development [59].

## 4. Mycotoxin Contamination in Fish Feed and Feed Ingredients

Plant proteins such as oilseeds are excellent alternatives to animal proteins in fish feeds because they are less expensive and are more abundant in many parts of the world [5]. Diets for Nile tilapia and warm water species such as carp and channel catfish are predominantly formulated using high amounts of grains and plant proteins, and as such, the feeds are at high risk to contamination by mycotoxins [60]. Cereals are common ingredients used in fish feeds and like the oilseeds, they are the main point of entry for many mycotoxins in humans and fish dietary systems, particularly in Africa [35]. Bran, which is also a common ingredient in fish feed, is usually derived from any cereal grains such as rice, maize, wheat, oats, barley, rye, and millet during the dry milling process [61]. Unfortunately, this dry milling is not likely to destroy mycotoxins. Mycotoxins are generally concentrated in the bran and outer layers of grains but are less in the endosperm [62]. This suggests that bran or wholemeal grains have the potential to contain higher concentrations of certain mycotoxins than those manufactured from flours or grits milled from the grain endosperm [61]. The use of mycotoxin contaminated bran and other ingredients provide an avenue for the finished fish feed to contain similar mycotoxins posing a health hazard in fish.

### 4.1. Occurrence of Aflatoxin

The incidences of aflatoxin contamination in fish feed have been reported in many countries of the world especially in the tropical and subtropical regions as shown in Table 2. Therefore, this means fish feed in both tropical and subtropical regions are more prone to aflatoxin contamination compared to the temperate regions [68]. The recommended regulatory limit for aflatoxin in fish feeds is 20 *μ*g·kg^−1^ [66], but a majority of samples from tropical countries are above this limit [35, 39, 40].

In the study of Marijani et al. [35], levels of mycotoxins in fish feeds and feed ingredients from fish farms, imported fish feeds, and feeds made by local feed millers in East Africa were analyzed. Results obtained revealed that aflatoxin contamination was higher in feed processed at farm level in terms of incidence rate (64.3%), feed ingredients (50%), and local commercial feed mills (35.7%), but not in imported feed. Inclusion of antifungal agents in imported feeds to prevent fungal growth during prolonged and varied storage conditions in farms might be a possible reason for the absence of aflatoxin in imported feed samples [35]. In the same study, fish feed samples from Kenya were found to be highly contaminated with aflatoxin at concentrations ranging from <2−806.9 *μ*g·kg^−1^, followed by those from Tanzania (<2–377.9 *μ*g·kg^−1^), Uganda (<2−28.0 *μ*g·kg^−1^), and Rwanda (<2–4.8 *μ*g·kg^−1^). In another study from Kenya, eighty-four percent of fish feed samples were tested positive for aflatoxins, ranging from 1.8 to 39.7 *μ*g·kg^−1^ [65]. Other studies from tropical countries, such as Brazil [39], Iran [40], and Egypt [44], reported that fish feeds were contaminated by aflatoxin at a concentration ranging from 1.83–67.35 *μ*g·kg^−1^, 0.46–68.5 *μ*g·kg^−1^, and 52.5–150 *μ*g·kg^−1^, respectively. In another study from Egypt, around 42.86% of fish feed were contaminated with aflatoxins at a value higher than the permissible limit of 20 *μ*g·kg^−1^ [69].

Alinezhad et al. [43] observed that pellets of rainbow trout feed and feed ingredients from Iran were contaminated with aflatoxins in the range of 1.83 to 67.35 *μ*g·kg^−1^. Altug and Beklevik [70] reported aflatoxin contamination in 56% of commercial fish feeds in Turkey is at levels of over 21 *μ*g·kg^−1^. Hassan et al. [44] studied the mycological quality of fish feed from Egypt and detected aflatoxin at significantly higher levels at mean levels of 105.2 ± 1.3 *μ*g·kg^−1^ in 40% of samples.

Gonçalves-Nunes et al. [37] screened raw materials and finished fish feeds for aflatoxin contamination in Brazil. Aflatoxin B_1_ was detected in the mean level of 1.1 *μ*g·kg^−1^, 7.4 *μ*g·kg^−1^, and 3.8 *μ*g·kg^−1^ in maize bran, other cereal products, and finished fish feed, respectively. Another fish feed survey conducted in Brazil by Hashimoto et al. [71] detected aflatoxin at maximum contamination levels of 15.6 *μ*g·kg^−1^. In addition, a survey on commercial shrimp feeds conducted in the Philippines found AFB_1_ contamination levels up to 120 *μ*g·kg^−1^ [69].

Aflatoxin contamination of cottonseed cake has been a major concern worldwide as extremely high contents ranging between 200 and 300 mg·kg^‒1^ were reported in samples exported from the USA to the European markets [72]. In a survey done by Rodriguez et al. [73] on contamination of aflatoxins in feeds and their ingredients in the Middle East and Africa, it was found out that sunflower meal has the highest contamination level in the whole survey (556 *μ*g·kg^−1^). A survey done in East Africa by Marijani et al. [35] found that cottonseed cake intended for fish feeds was contaminated by aflatoxin with a maximum concentration of 377.9 *μ*g·kg^−1^, while soybeans were not contaminated. Sunflower seed cake was the only ingredient that contained the highest AFB_1_ concentration of 806.9 *μ*g·kg^−1^ when compared to maize bran, soybeans, cottonseed cake, and rice bran [35]. In another survey from Tanzania conducted by Mmongoyo et al. [74], sunflower seed cake was contaminated with aflatoxin with a maximum concentration of 662.7 *μ*g·kg^−1^ recorded. Interventions to control aflatoxin contamination along the oilseed product value chain should be implemented to enhance feed safety in African countries.

Looking at the data in Table 2, it can be confirmed that the occurrence of aflatoxin is very high from fish feeds from Africa. This is worrying as the high incidences of aflatoxicosis in human were also reported in Africa [75]. During human aflatoxicosis outbreaks in 2005 and 2006, maize were heavily contaminated with aflatoxin with maximum levels of 48,000 and 24,000 *μ*g·kg^−1^, respectively [75] Daniel et al. [75] concluded that drought and famines followed by unseasonable rains during harvest and improper storage of homegrown maize in moist conditions were the reasons behind the high incidence of aflatoxicosis.

Maize intended for fish feeds was contaminated by AFB_1_ at a maximum concentration of 135 *μ*g·kg^−1^ [35]. Similar results were also reported by Reddy and Salleh [76], who found out that 22.5% of samples of maize had AFB_1_ contamination ranging from 20.6 to 135 *μ*g·kg^−1^. The poorest quality maize is used for animal feeding, which makes animals more at risk to aflatoxicosis [45].

Rice is another important staple food in Africa and Asia and its bran is widely used for animal feeding [77]. Rice bran intended for fish feed from East Africa were not contaminated by aflatoxin [35]. In another study from Iran, rice bran was contaminated by aflatoxin with the mean concentration of 18 *μ*g·kg^−1^ [78]. Wheat does not grow well in tropical climates; however, its bran is widely used as a component of animal and fish feeds [28, 79]. Another cereal which is used in fish feed as an ingredient is sorghum. The incidence of aflatoxin in sorghum and millet from northern Nigeria was investigated by Apen Daneil et al. [80], and they found out that 28.6% sorghum (0.96–21.74 *μ*g·kg^−1^) and millet grain (105–14.96 *μ*g·kg^−1^) were contaminated with aflatoxin. Out of 52 ingredients intended for fish feed from East Africa, sorghum and wheat bran were detected at a very low concentration of less than 3 *μ*g·kg^−1^ [35, 81]. Another study from Brazil reported that out of 140 sorghum collected, only 12.8% were contaminated by aflatoxin [82]. Shetty and Bhat [83] found out that 20% of normal sorghum and 89% of normal maize samples also contained aflatoxin B_1_. Bandyopadhyay et al. [84] suggested that if the primary cereal is sorghum instead of maize, then the risk of aflatoxin-related problems is reduced by 4-fold. Low level of aflatoxin contamination in wheat bran, sorghum, and soya bean suggests that they are likely to be useful in the formulation of fish feeds with aflatoxin below levels that could elicit any adverse complications on fish health.

### 4.2. Occurrence of *Fusarium* Mycotoxins

There are several reports on the contamination of cereal grains and animal feed with *Fusarium* mycotoxins worldwide [17, 79]. The most important among them are the trichothecenes, ZEN, and the fumonisins [85]. The trichothecenes are subdivided into four basic groups, with types A and B being the most important. Type A trichothecenes include T-2 toxin, HT-2 toxin, neosolaniol, and diacetoxyscirpenol (DAS) [85]. Type B trichothecenes include DON also known as vomitoxin, nivalenol (NIV), and fusarenon-X. Fumonisins, particularly FB_1_, are found in maize grain which is a major component of feeds for warm water fish [86]. Contamination of fumonisin in cereals is dependent on the geographical region, season, and conditions under which the particular cereal is grown, harvested, and stored [45]. The prevalence of fumonisin has been reported to be 100% or close to it in all surveillance studies on maize from different parts of Africa [13]. Several studies have shown that maize bran which is mainly used for animal/fish feed has been contaminated with FBs [21, 79]. Fumonisin was detected at the concentration of 1 mg·kg^−1^ on the maize bran used for animal feed from Tanzania [87]. The prevalence of *F. verticillioides* and production of FB_1_ in cereal grains and oilseeds in Zimbabwe was established by Gamanya [88]. While the authors did not find *Fusarium* and FB_1_ contamination in sunflower and soybean samples tested, high incidences were recorded for maize followed by wheat and sorghum [88]. Maize bran and soybeans from East Africa used for fish feeds contained a maximum of up to 3970.1 *μ*g·kg^−1^ and 1402.3 *μ*g·kg^−1^ of FB_1_, respectively, while cottonseed cake, sunflower seed cake, and rice bran were not contaminated with FB_1_ [35]. This suggests that maize is more susceptible to FBs when compared to other feed ingredients. In the same study, they found out that fish feeds processed at the farm level contained a maximum FBs concentration of 2834.6 *μ*g·kg^−1^ [35], and samples tested were below the regulatory limits of 5000 *μ*g·kg^−1^ recommended by EU [67].

DON is the most often occurring trichothecene and is prevalent in crops used for food and feed production, generally found in various cereal crops such as wheat, barley, oats, rye, rice, and maize [85]. Natural occurrence of DON in cereals is certainly prevalent, and surveys from South America, Canada, China, and many countries of Europe have shown contamination levels in excess of 50% in oats, barley, and wheat with mean concentrations as high as 9 mg·kg^‒1^ in barley [46]. In a survey carried out between 2004 and 2007, DON was the predominant mycotoxin with highest levels detected in wheat bran [89]. Few studies have been carried out on the contamination of DON in finished fish feeds [86]. A survey done in Central Europe has shown that more than 80% of the samples from commercial fish feed were contaminated with DON with a mean concentration of 289 *μ*g·kg^−1^ recorded [64]. Fish feed processed at farm level were contaminated with the mean DON concentration of 755 *μ*g·kg^−1^ while among the ingredients, maize bran was highly contaminated with 984 *μ*g·kg^−1^ [35]. Another study from Nigeria found out that fish feeds were contaminated with a mean DON concentration of 85.9 *μ*g·kg^−1^ [90]. All fish feeds from these studies were below the regulatory limits of 5000 *μ*g·kg^−1^ recommended by EU [67]. Sixty-eight samples of shrimp and fish feed from Asia and Europe were contaminated with DON at a mean concentration of 162 *μ*g·kg^−1^ and maximum level of 413 *μ*g·kg^−1^ [91].

Zearalenone, a toxic metabolite of *Fusarium* fungi is commonly found as contaminant in maize, and also it may occur in oats, barley, wheat, and sorghum [92]. However, the production of ZEN is favored by high humidity and low temperature conditions [93]. It may co-occur with DON in grains such as wheat, barley, oats, and maize and FBs in maize [92]. ZEN was found in fish feed from Asia with average concentrations of 76.2 *μ*g·kg^−1^ [28]. Other studies from Europe reported that fish feeds were contaminated with ZEN with a maximum concentration of 511 *μ*g·kg^−1^ [64]. However, the ZEN values found in these studies do not exceed the values (5000 *μ*g·kg^−1^) currently recommended by the European Commission in animal feeds [67]. DON and ZEN in unprocessed cereals and soybean were detected at the mean concentrations of 1,461 ± 2,265 *μ*g·kg^−1^ and 656 ± 853 *μ*g·kg^−1^, respectively, in samples collected in 2014, while in 2015 these means were 2,687 ± 2,731 *μ*g·kg^−1^ and 1,140 ± 1,630 *μ*g·kg^−1^, respectively [94]. The authors suggested that higher contamination determined during 2015 could be explained by high to extreme humidity evidenced in the period of cereals' growth and harvesting. The occurrence of DON and FBs in fish feeds, even at low levels, may be of concern, since it can cause growth retardation and immunotoxic effects in fish [95]. These results suggest that ZEN contamination may pose little health risk (if any) to the consumers of the fish [96].

### 4.3. Other Mycotoxins

Other mycotoxins like OTA, NIV, DAS, T−2 toxin, alternariol (AOH), and ROQ-C have been reported to occur in fish feeds and ingredients intended for fish feed formulation [35]. Cottonseed cake for fish feed formulation was the only ingredient contaminated by OTA with a maximum concentration of 24.2 *μ*g·kg^−1^ [35]. Nivalenol was detected in fish feeds processed at farm level with a maximum concentration of 732.5 *μ*g·kg^−1^, while no ingredients intended for fish feeding was contaminated by NIV. Other mycotoxins like DAS, T-2, and ROQ-C were detected in fish feeds and their ingredients but at very low concentrations [35].

Also, an immunosuppressive mycotoxin, gliotoxin, was detected in oilseed cakes at levels up to 45 *μ*g·kg^−1^, which was associated with the presence of toxigenic isolates of *A. fumigatus* [97].

## 5. Effects of Mycotoxin on Fish Health

The toxic effects of mycotoxins are not only depended on the dose in feeds but as well as on the duration of toxin exposure, species, as well as the sex and age of the animal [94].

Among the mycotoxins, AFB_1_ is the most studied in fish, possibly because of its natural occurrence being most widely found in tropical countries and that it is a known human carcinogen and most potent hepatotoxin [98]. These studies on toxicity effect of AFB_1_ on farmed aquatic species include rainbow trout, [23, 99, 100]; Nile tilapia, [14, 19, 101–104]; channel catfish, [22, 105]; rohu, [106, 107]; sea bass, [98]; gibel carp, [18]; beluga, [108]; and abalone [63].

Considering the results of the studies in Table 3, the biological effects of AFB_1_ in these aquatic species depend on the toxin's concentration in feed and species. Channel catfish, *Ictalurus punctatus*, appears to be one of the most resistant among fish species when exposed to AFB_1_, while rainbow trout is the most sensitive to AFB_1_, and exposing them at concentrations as low as 0.4 *μ*g AFB_1_ kg^−1^ may cause a 14% chance of developing tumors [22, 99]. Previously, there are no studies on hepatocellular carcinomas in channel fish caused by AFB_1_, but there are reports that dosing them with higher concentrations of AFB_1_ resulted in decreased growth rate and moderate internal lesions [22]. European sea bass is also sensitive to AFB_1_; El-Sayed and Khalil [98] found that exposing them for 4 days with median lethal concentration (LC50) of 180 *μ*g kg^−1^ AFB_1_ causes aflatoxicosis. Other studies on fish have shown reduced growth rates particularly on Nile tilapia and channel catfish-fed diets containing 1880 and 10000 *μ*g AFB_1_ kg^−1^ feed, respectively [14, 17, 22]. The mortality rate of 17% was reported in Nile tilapia fed diets containing 2000 *μ*g AFB_1_ kg^−1^ [101]. Aflatoxin is also known to affect eye opacity resulting in cataract and blindness, yellowing of the body surface, wounds on the body surface, fin and tail rot, abnormal swimming, feeble and stationary movements, and reduced appetite in tilapia-fed aflatoxin-contaminated diet [19].

Aflatoxin has been reported to disrupt the reproductive system in both male and female animals; however, very few studies had been reported in aquatic animals [116]. The few existing studies show a significant decrease in ovary weight, fecundity, and egg size of gibel carp fed on AFB_1_-treated ration [18]. In Nile tilapia, a negative effect on gonadosomatic index, fecundity, sperm count, sperm activity, and serum estradiol-17β concentrations was observed after feeding them with 1 and 3 mg·kg^−1^ AFB_1_ contaminated for 3 months [117].

The contamination values of AFs found in fish feeds and their ingredients from Africa were high (Table 2) and, as shown in Table 3, can negatively affect farmed fish, thus leading to economic loss to fish farmers.

*Fusarium* mycotoxins are able to induce both acute and chronic toxic effects. Previous studies have shown that these effects depend on the dose, duration of exposure, and fish species that are exposed (Table 4). Rainbow trout are sensitive to DON when exposed at low dose, while channel catfish are much less responsive (Table 4). Hooft et al. [100] reported that feeding rainbow trout with low, graded levels of DON ranging from 3.0 × 10^−4^ to 2.6 × 10^−3^ mg·kg^−1^ from naturally contaminated maize resulted in highly significant decrease in growth, feed intake, feed efficiency, and protein and energy utilization, whereas channel catfish fed of diets containing up to 10 mg·kg^−1^ DON from either a purified source or naturally contaminated wheat had no effect on the feed consumption, growth, hematocrit values, or liver weight [119]. Also, the rainbow trout liver is sensitive to FB_1_ because it induces changes in sphingolipid metabolism [131] and is a cancer promoter in this species [123]. Growth performance of Nile tilapia fingerlings was negatively affected when fed with both moniliformin (MON) and FB_1_ at 70 and 40 mg·kg^−1^, respectively. However, when compared to channel catfish, Nile tilapia appears to be more resistant to these two mycotoxins as no mortality and histopathological lesions have been reported [102].

Reduction in growth, feed efficiency, and feed intake in fish fed with DON-contaminated diet was reported by Tola et al. [95], Hooft et al. [100], and Döll et al. [118]; however, there are also reports of DON not interfering in the weight gain of fish [132, 133].

ZEN has been implicated in reproductive disorders of farm animals [134]; however, very few studies have been done on the effect of ZEN in farmed aquatic species. In zebrafish (*Danio rerio)*, ZEN reduced spawning frequency [126] or changed their relative fecundity from one generation to another [127]. In another study, defects in the heart and eye development and upward curvature of the body axis of were observed when zebrafish larvae were exposed to 500 *µ*g·L^−1^ or higher of ZEN [135]. By considering the few studies made in aquaculture species (Table 4), we would presume that ingestion of ZEN may affect growth performance, but it depends on species, dose, and duration of exposure, and it can result in complications in broodstocks of farmed species and monosex-cultured species.

Scarce information is available on the toxicity of OTA in aquatic species. A significant reduction in weight gain, poorer feed conversion rate, lower survival, and hematocrit was observed in channel catfish fed with OTA-contaminated diets [111]. Furthermore, moderate-to-severe histopathological lesions of the liver and posterior kidney were observed [113]. While a significant decrease in erythrocyte count (RBCs), haemoglobin content (Hb), and haematocrit value (Hct) in Nile tilapia exposed to low OTA level (400 *µ*g·kg^−1^) was seen, in Nile tilapia exposed to 600 *µ*g·kg^−1^ diet mean corpuscular volume (MCV), mean corpuscular haemaglobin (MCH), and mean corpuscular haemaglobin concentrate (MCHC) blood indices significantly reduced [136]. In addition, as shown in Table 4, OTA has a negative impact on shrimp even after feeding them with 1000 *µ*g·kg^−1^ contaminated diet for 8 weeks. However, increasing the dose and exposure duration might affect the shrimp negatively.

Other mycotoxins like sterigmatocystin, which is closely related to the aflatoxin as a precursor in aflatoxin biosynthesis and carcinogenic, have been studied in Nile tilapia [115]. Stg has genotoxic and toxicopathological effects in Nile tilapia, [115]. Stg also decreases body weight and increase in frequencies of micronucleated red blood cells (MN RBC) and chromosomal aberrations in the kidney of Nile tilapia [114]. Studies on the effects of ROQ-C on animals and fish are limited; however, CPA has been reported to cause anorexia, diarrhea, pyrexia, dehydration, weight loss, ataxia, immobility, and extensor spasm at the time of death in several animals [33].

As shown in Tables 3 and 4, different studies highlight that mycotoxins are a serious problem to farmed aquatic species. A majority of these experiments were conducted based on chronic character, contaminated feed as the route of exposure, and several different doses. It is important to maintain standards when performing the experiments in relation to sex, as studies show that male animals are more susceptible to mycotoxin [137], divide the toxicology tests on acute, subchronic, and chronics, and also consider species, age, rearing conditions, route of exposure, and dose according to each type of mycotoxins, mainly to detect toxic effects on fish.

## 6. Synergistic Effect of Mycotoxins in Fish

Mycotoxins often occur concurrently [40]. Thes multiple mycotoxin contaminations in fish feeds are crucial as mycotoxins may toxicologically interact with each other eliciting marked synergistic and additive actions [138] especially between mycotoxins found at high concentrations. This might increase the negative impact of mycotoxins in farmed aquatic species at lower levels than when present in single contamination. There are limited data on toxic effects of mycotoxin mixtures in farmed aquatic species [123]. FB_1_ was not carcinogenic when rainbow trout was fed at 0, 3.2, 23, or 104 mg·kg^−1^ FB_1_ for a total of 34 weeks. But trout-fed FB_1_ (≥23 mg·kg^−1^ FB_1_ for 42 weeks) promoted AFB_1_-initiated liver tumors; this result also suggests the importance of long-term contamination as a factor influencing the susceptibility of animals. He et al. [139] examined the individual and synergistic effect of DON and AFB_1_ on primary hepatocytes of common carp and inferred that the toxic effects of the combined mycotoxins were greater than the effects of single mycotoxins. McKean et al. [140] examined the synergistic effects of AFB_1_ and T-2 toxin in mosquitofish (*Gambusia affinis*), showing a significant additive interaction in the toxic response to the combination of mycotoxins. The co-occurrence of AF and OTA presents a health risk in fish because of their synergistic and/or additive effects [141]. Critical is that these mycotoxins can potentially be carried over to human food of animal origin and may cause public health threats [141]. To the best of our knowledge, very few studies were carried out for investigating the interacting effects of multimycotoxins in farmed aquatic animals; however, considering the previous studies carried out for other animals [142–144], we could consider that mycotoxin interactions may likewise affect negatively farmed aquatic species.

## 7. Conclusion

This review shows that fish feeds and their ingredients are frequently contaminated with mycotoxigenic fungi and mycotoxins. Ingredients, especially grains and oilseeds, are the main reservoirs for mycotoxins in fish feed. Ingestion of mycotoxin-contaminated feeds can affect fish health and also contributes to the economic loss of the fish farmers. Also, attention should be taken on the possible carryover of mycotoxins from feed ingredients and finished feed to the meat of farmed aquatic animals for human consumption. Hence, it is important to ensure the control of mycotoxin contamination in fish feeds and their ingredients. Use of seed coat with the small, compact grains (wheat, rice, oat, and sorghum) and those encapsulated in hard seed coats (beans and soybeans) might reduce the contamination of mycotoxin in feeds since they are less susceptible to fungal infection and mycotoxin formation than larger grains such as maize. Since mycotoxins are also produced during storage conditions, it will be important to monitor routinely raw materials as well as finished feeds. Awareness and sensitization on proper storage facilities, duration, and condition for feeds and ingredients are recommended to farmers. Antifungal agents should be used to reduce mycotoxin contamination in feed ingredients and finished fish feeds. Further research is needed to test the synergistic effects of mycotoxins when diets are contaminated with more than one mycotoxin.

## Figures and Tables

**Table 1:** Frequently isolated fungi from fish feeds and feed ingredients from previous studies.

Source	Country	Sample size	Common isolates	Frequency of isolation (%)	References
Tilapia feeds	Egypt	25	*A. flavus* ^*∗*^	48	Mohammed et al. [34]
			*A. niger*	40	
			*A. fumigatus*	8	
			*A. ochraceus*	4	
			*Penicillium* sp.,	40	
			*Cladosporium* sp.	8	
			*Candida* sp.	40	

Fish feeds and ingredients	East Africa	52	*A. flavus* ^*∗*^	54.5	Marijani et al. [35]
			*A. tamarii*	9.1	
			*Mucor *sp.	9	
			*Phoma* sp.	6.1	
			*A. niger*	6	
			*E.rubrum*	3	
			*P. chrysogenum*	3	

Commercial and formulated fish feeds	Kenya	121	*Asperigillus* sp.	50.5	Njagi [36]
			*Mucor* sp.^*∗*^	56	
			*Rhizopus* sp.	49.5	
			*Saprolegnia* sp.	42.5	
			*Penicillium* sp.	31	

Fish feeds and ingredients	Brazil	54	*Penicillium* sp.^*∗*^	83.3	Gonçalves-Nunes et al. [37]
			*Aspergillus* sp.	66.7	
			*Rhizopus* sp.	23.3	
			*Cladosporium* sp.	20	

Rainbow trout feed	Argentina	28	*Cladosporium cladosporioides* ^*∗*^	53.6	Greco et al. [38]
			*Eurotium repens*	21.4	
			*Eurotium rubrum*	14.3	
			*Mucor *sp.	7.1	
			*A. versicolor*	3.6	
			*P. crustosum*	3.6	
			*P. expansum*	3.6	
			*P. chrysogenum*	3.6	

Fish feeds	Iran	86	*A. flavus* ^*∗*^	47.3	Fallah et al. [39]
			*A. parasiticus* ^*∗*^	16.1	
			*A. niger*	13.9	
			*A. ochraceus*	10.2	
			*A. fumigates*	9	
			*A. versicolor*	<5	
			*A. carbonarius*	<5	
			*A. nomius*	<5	
			*A.ustus*	<5	
			*Fusarium* sp.	26.2	
			*Eurotium* sp.	10	
			*Penicillium* sp.	41.5	
			*Mucor* sp.	<10	
			*Cladosporium* sp.	<5	
			*Alternaria* sp.	<5	

Fish feed	Brazil	60	*Cladosporium* sp.^*∗*^	85	Barbosa et al. [40]
			*P. citrinum* ^*∗*^	71	
			*A. flavu*	35	
			*A. niger*	36	
			*Eurotium* sp.	20	
			*Wallemia*	40	
			*Aureobasidium*	10	
			*Mucor, and*	10	
			*Nigrospora* sp.	<10	
Fish feeds	Brazil	36	*A. flavus* ^*∗*^	60.47	Filho et al. [41]
			*A. fumigates*	6.98	
			*A. terreus*	2.32	
			*A. candidus*	2.32	
			*A. oryzae*	6.98	
			*A. penicillioides*	2.32	
			*Eurotium* sp.	18.61	
			*Penicillium* sp.	19.18	
			*Cladosporium* sp.	16.44	
			*Fusarium* sp.	8.22	

Tilapia feed	Mexico	30	*Aspergillus flavus* ^*∗*^	6.7	Rodriguez–Cervantes et al. [42]
			*Fusarium* sp.	6.7	

Sea bass feeds	Portugal	87	*Aspergillus niger* ^*∗*^	39.1	Almeida et al. [10]
			*Aspergillus glaucus*	29.9	
			*Penicillium* sp.	28.7	
			*Cladosporium* sp.	28.7	
			*Fusarium* sp.	25.3	

Rainbow trout feed	Iran		*A. flavus* ^*∗*^	60.66	Alinezhad et al. [43]
			*A. niger*	19.67	
			*A. ochraceus*	1.0	
			*A. fumigates*	2.0	
			*A. clavatus*	1.0	
			*Penicillium* sp.	14.0	
			*Absidia* sp.	12.0	
			*Mucor* sp.	2.0	
			*Alternaria* sp.	2.0	

Fish feeds	Egypt	50	*A. flavus* ^*∗*^	56.0	Hassan et al. [44]
			*A. niger*	4.0	
			*A. ochraceus*	2	
			*A. candidus*	8.0	
			*Penicillium* sp.	22.0	
			*Mucor* sp.	50.0	
			*Cladosporium* sp.	2.0	
			*Rhizopus* sp.	4.0	

^*∗*^The most commonly isolated species.

**Table 2:** Mycotoxin levels (*μ*g·kg^−1^) in fish feed from developing countries.

Source	Country	Sample size	Mycotoxin contamination^*∗*^								PL %^+^	Reference
			AF	FB	DON	OTA	NIV	AOH	T-2	ZEN		
Tilapia feeds	Egypt	25	<LOD	na	na	na	na	na	na	na	0	Mohamed et al. [34]
Fish feeds processed at farm level	East Africa	52	126	755.4	2834.6	nd	732.5	91.3	<LOD	na	48 (AF), 0 (FB), 0 (DON)	Marijani et al. [35]
Abalone feeds	South Africa		0.98	424	100	0.259	100	na	na	na	0	Laubscher [63]
Tilapia feeds	Mexico	30	<LOD	2587	na	na	na	na	na	na	0	Rodriguez-Cervantes et al. [42]
Rainbow trout	Argentina	28	2.82^*∗∗∗*^	<LOD	230^*∗∗*^	5.26^*∗∗*^	na	na	70.08^*∗∗*^	87.97^*∗∗*^	0	Greco et al. [38]
Fish feeds	Iran	86	68.5	na	na	na	na	na	na	na	17.1	Fallah et al. [39]
Fish feeds	Brazil	60	<LOD	4.94	na	<LOD	na	na	na	na	0	Barbosa et al. [40]
Fish feeds	Central Europe	na	na	na	825	na	na	na	na	511	0	Pietsch et al. [64]
Fish feeds	Egypt	50	150	na	na	na	na	na	na	na	40	Hassan et al. [44]
Fish feeds	Brazil	54	3.8^*∗∗∗*^	na	na	na	na	na	na	na	0	Gonçalves-Nunes et al. [37]
Sea bass feeds	Portugal	87	nd	na	na	na	na	na	na	na	0	Almeida et al. [10]
Rainbow trout	Iran		67.35	na	na	na	na	na	na	na	nm	Alinezhad et al. [43]
Fish feeds	Kenya	81	39.7	na	na	na	na	na	na	na	13.5	Mwihia et al. [65]

^*∗*^Maximum level of mycotoxin content in positive samples; ^*∗∗*^median of positive samples excluding results of estimated concentrations; ^*∗∗∗*^mean level of mycotoxin content in positive samples; ^+^percentage of samples that are above the permissible limit (PL) of AFs, FBs, and DON in feeds recommended by FDA [66] and EU [67]; nd, not detected; na, not analyzed in the study; nm, not mentioned in the study.

**Table 3:** Toxic effects of aflatoxins, ochratoxin A, and sterigmatocystin in different fish species.

Mycotoxin	Species	Exposure dose	Administration	Duration of exposure (weeks)	Toxicity effect	References
Aflatoxin	Nile tilapia *(Oreochromis niloticus)*	100 *μ*g AFB_1_/kg	Feed: oral	10	Reduced the growth	El-Banna et al. [101]
	Nile tilapia	200 *μ*g AFB_1_/kg	Feed: oral	10	Mortality (16.7%)	El-Banna et al. [101]
	Nile tilapia	5–38.62 *μ*g AFB_1_/kg	Feed: oral	10	Survival rate reduced by up to 67%	Cagauan and Tayaban [19]
	Nile tilapia	29 *μ*g AFB_1_/kg	Feed: oral	10	Yellowing of the body surface	Cagauan and Tayaban [19]
	Tilapia	200 ppb AFB_1_/kg	Feed: oral	10	Total erythrocyte count, total leucocyte count, and hemoglobin count decreased; weight gain lowest and reduction in the rate survival rate	Selim et al. [104]
	Tilapia	793 and 1641 *μ*g AFB_1_/kg	Feed: oral	5	Yellowing of the body surface	Deng et al. [103]
	Tilapia	793 and 1641 *μ*g AFB_1_/kg	Feed—oral	15	Darkening of body surface	Deng et al. [103]
	Tilapia	2.5 mg AFB_1_/kg	Feed: oral		Affect the hematocrit and growth performance	Tuan et al. [109]
	Tilapia	2.5 mg AFB_1_/kg	Feed: oral	20	Abnormal behavior	Deng et al. [103]
	Tilapia	245 *μ*g AFB_1_/kg	Feed: oral	20	Feed efficiency rate decreased	Deng et al. [103]
		245, 638, 793 and 1641 *μ*g AFB_1_/kg	Feed: oral	20	Weight gain lowest	Deng et al. [103]
	Channel catfish *(Ictalurus punctatus)*	10000 *μ*g AFB_1_/kg	Feed: oral	10	Decreased leukocyte count, increased haematopoietic activity of blood-forming tissues	Jantaroai and Lovell [22]

Aflatoxin	Rohu (*Labeo rohita)*	2.50 and 5.00 mg·kg^−1^	Intraperitoneal (i.p.)	10	Reduction in production of oxygen radicals by neutrophils	Sahoo and Mukherjee [110]
	Rohu	1.25; 2.50 and 5.00 mg·kg^−1^	Intraperitoneal (i.p.)	10	Reduction of total protein and globulin levels	Sahoo and Mukherjee [110]
	Rohu	10, 20 and 40 mg·kg^−1^	Feed: oral	8	Total erythrocyte count, total leucocyte count, hemoglobin count, and nitroblue tetrazolium decreased	Mohapatra et al. [107]
	Gibel carp (*Carassius auratus gibelio)*	20, and 2000 *μ*g AFB_1_ kg^−1^	Feed: oral	24	Gonadosomatic index (GSI), absolute brood amount (AF), relative brood amount (RF), and oocyte diameter were significantly lower	Huang et al. [18]
	Juvenile rainbow trout	1190 *μ*g·kg^−1^	Feed: oral	3	Mortality	Nomura et al. [99]
	Sea bass (*Dicentrarchus labrax* L.)	0.18 mg·kg^−1^	Feed: oral	4 days	Loss of equilibrium, rapid opercular movement, and hemorrhages of the dorsal skin surface	El-Sayed and Khali [98]
	Sea bass	0.018 mg·kg^−1^	Feed: oral	6	^*∗*^ALT, AST, and ALP enzymes increased; total protein; Albumin; and Globulin increased	El-Sayed and Khalil [98]
OTA	Channel catfish	1.0, 2.0, 4.0, or 8.0 mg·kg^−1^	Feed: oral	8	Reductions in body weight gain	Manning et al. [111]
		4.0, or 8.0 mg·kg^−1^	Feed: oral	8	Feed conversion ratio was significantly poorer	Manning et al. [111]
		8.0 mg·kg^−1^	Feed: oral	8	Hematocrit was significantly lower	Manning et al. [111]

OTA	Juvenile common carp	4.0 mg·kg^−1^	Feed: oral	6	Mortality (80.49%)	Manning et al. [112]

OTA	Black tiger shrimp (*Penaeus monodon)*	1000 μg·kg^−1^	Feed: oral	8	No negative impact in shrimp	Supamattaya et al. [113]

Stg	Nile tilapia	5, 10 and 50 *μ*g·ml^−1^	Intragastric	4	Clastogenic, decrease of body weight, and the increase in frequencies of micronucleated red blood cells (MN RBC) and chromosomal aberrations in the kidney	Abdel-Wahhab et al. [114]

Stg	Nile tilapia	1.6 *μ*g·kg^−1^ bwt	Corn oil: oral	4	Genotoxic and toxicopathological effects	Mahrous et al. [115]

^*∗*^ALT: alanine aminotransferase; ALP: alkaline phosphatase; AST: aspartate transaminase.

**Table 4:** Toxic effects of *Fusarium* mycotoxins in different species of fish.

Mycotoxin	Species	Exposure dose	Administration	Duration of exposure (weeks)	Toxicity effect	References
DON	Rainbow trout *Oncorhynchus mykiss*	2.6 mg·kg^−1^	Feed: oral	8	Decrease in growth, feed intake, feed efficiency, and protein and energy utilization.	Hooft et al. [100]
	Atlantic salmon *Salmo salar* L.	3.7 × 10^−3^ mg·kg^‒ 1^		8	Reduction in feed intake and decrease in specific growth rate	Döll et al. [118]
	Channel catfish *Ictalurus punctatus*	5.0–10.0 mg·kg^−1^	Feed: oral	8	Mortality	Manning et al. [119]

T-2 toxin	Juvenile channel catfish	1.25, 2.5, and 5.0 mg·kg^−1^	Feed: oral	8	Reductions in growth and hematocrit values were adversely affected	Manning et al. [120]
		5.0 mg·kg^−1^	Feed: oral	8	Histopathological anomalies of stomach, head, and trunk kidneys	Manning et al. [120]
	Juvenile common carp	1.0 or 2.0 mg·kg^−1^	Feed: oral	6	Mortality	Manning et al. [112]
	Pacific white shrimp *Litopenaeus vannamei*	1.2, 2.4, 4.8, and 12.2 mg·kg^−1^	Feed: oral	3	Decrease in growth and survival rate	Deng et al. [103]
		2.4 and 4.8 mg·kg^−1^	Feed: oral	3	Antioxidant enzymes, superoxide dismutase (SOD) and glutathione peroxidase (GPx), total antioxidant capacity (T-AOC), and also glutathione (GSH) content increased	Deng et al. [103]
		12.2 mg·kg^−1^	Feed: oral	3	SOD and GPx, T-AOC, and GSH content decreased, cell autophagy	Deng et al. [103]
MON	Channel catfish	20, 40, 60, and 120 mg·kg^−1^	Feed: oral	10	Reductions in growth,	Yildirim et al. [121]
	Channel catfish	60 mg·kg^−1^	Feed: oral	10	Low hematocrit level and high serum pyruvate level	Yildirim et al. [121]
	Nile tilapia	60 and 150 mg·kg^−1^	Feed: oral	8	Reductions in growth and high serum pyruvate levels	Tuan et al. [122]
	Nile tilapia	150 mg·kg^−1^	Feed: oral	8	Hematocrit was significantly low	Tuan et al. [122]

FB_1_	Channel catfish	80, 320, or 720 mg·kg^−1^	Feed: oral	14	Reductions in growth, lower hematocrit and red cell counts, and higher white cell counts	Lumlertdacha et al. [105]
	Channel catfish	20, 80, 320, or 720 mg·kg^−1^	Feed: oral	14	Swollen hepatocytes in the liver with lipid-containing vacuoles, lymphocyte infiltration, and scattered necrotic hepatocytes	Lumlertdacha et al. [105]
	Rainbow trout	23 mg·kg^−1^	Feed: oral	42	Cancer promoter	Carlson et al. [123]
	Nile tilapia	40, 70, 150 mg·kg^−1^	Feed: oral	8	Lower mean weight gains	Tuan et al. [122]
	Nile tilapia	150 mg·kg^−1^	Feed: oral	8	Haematocrit was decreased and ratio between free sphinganine and free sphingosine (SA/SO) in the liver increased	Tuan et al. [122]
	Common carp *Cyprinus carpio*	100 and 10 mg·kg^−1^	Feed: oral	6	Blood vessels, liver, exocrine and endocrine pancreas, excretory and haematopoietic kidney, and heart and brain were sensitive	Petrinec et al. [124]
	Common carp	0.5 and 5.0 mg·kg^−1^	Feed: oral	6	Loss of body weight and alterations of haematological and biochemical parameters in target organs	Pepeljnjak et al. [125]
		5.0 mg·kg^−1^	Feed: oral	6	Increase in bacterial infection	Pepeljnjak et al. [125]

ZEN	Zebrafish *Danio rerio*	1000 and 3200 ng·L ^−1^		6	Reduced spawning frequency	Schwartz et al. [126]
	Zebrafish	1000 ng·L^−1^		26	Affect growth and changed relative fecundity from one generation to another	Schwartz et al. [127]
	Black tiger shrimp *Penaeus monodon Fabricius*	500 and 1000 mg·kg^−1^	Feed: oral	10	Histological changes in hepatopancreatic tissue	Bundit et al. [128]
	Common carp	0.332, 0.621 and 0.797 mg·kg^‒1^	Feed: oral	4	No effect on growth but effects on haematological parameters	Pietsch et al. [129]
	Juvenile rainbow trout	1.810 mg·kg^−1^	Feed: oral	10	No effects on growth and may accelerate sexual maturation of female fish	Woźny et al. [130]
